# Medium-Dose Chronic Cannabidiol Treatment Reverses Object Recognition Memory Deficits of *APP*_*Swe*_ */PS1ΔE9* Transgenic Female Mice

**DOI:** 10.3389/fphar.2020.587604

**Published:** 2020-12-04

**Authors:** Madilyn Coles, Georgia Watt, Fabian Kreilaus, Tim Karl

**Affiliations:** ^1^ School of Medicine, Western Sydney University, Campbelltown, NSW, Australia; ^2^ Neuroscience Research Australia, Randwick, NSW, Australia

**Keywords:** Alzheimer’s disease, *APP*_*Swe*_*/PS1ΔE9*, transgenic mouse model, cannabidiol, treatment, behavior

## Abstract

Alzheimer’s disease (AD) is a neurodegenerative disease that causes behavioral and cognitive impairments. The phytocannabinoid cannabidiol (CBD) has anti-inflammatory, antioxidant, and neuroprotective properties, and *in vitro* and limited *in vivo* evidence suggests that CBD possesses therapeutic-like properties for the treatment of AD. Cannabinoids are known to have dose-dependent effects and the therapeutic potential of medium-dose CBD for AD transgenic mice has not been assessed in great detail yet. 12-month-old control and *APP*_*Swe*_ */PS1ΔE9* (*APPxPS1*) transgenic female mice were treated daily via intraperitoneal injection with 5 mg/kg bodyweight CBD (or vehicle) commencing three weeks prior to the assessment of behavioral domains including anxiety, exploration, locomotion, motor functions, cognition, and sensorimotor gating. *APPxPS1* mice exhibited a hyperlocomotive and anxiogenic-like phenotype and had wild type-like motor and spatial learning abilities, although AD transgenic mice took generally longer to complete the cheeseboard training (due to a lower locomotion speed). Furthermore spatial learning and reversal learning was delayed by one day in *APPxPS1* mice compared to control mice. All mice displayed intact spatial memory and retrieval memory, but *APPxPS1* mice showed reduced levels of perseverance in the cheeseboard probe trial. Importantly, vehicle-treated *APPxPS1* mice were characterized by object recognition deficits and delayed spatial learning, which were reversed by CBD treatment. Finally, impairments in sensorimotor gating of *APPxPS1* mice were not affected by CBD. In conclusion, medium-dose CBD appears to have therapeutic value for the treatment of particular behavioral impairments present in AD patients. Future research should consider the molecular mechanisms behind CBD’s beneficial properties for AD transgenic mice.

## Introduction

Alzheimer’s disease (AD) is an insidious neurodegenerative disease that is caused by progressive damage to neuronal cells and results in irreversible cognitive and behavioral deficits including memory loss, spatial disorientation, and language impairments. AD is the most common form of dementia and is currently incurable and without effective preventative options and usually leads to death due to secondary diseases such as pneumonia (Burns et al., 1990; Brunnström and Englund, 2009). Often a verified diagnosis of AD can only be made postmortem, with the two main pathological hallmarks of AD being (1) the extracellular accumulation of amyloid-beta (Aβ) protein fragments around the neurons in the brain, forming Aβ plaques, and (2) the intracellular accumulation of hyperphosphorylated microtubule-associated protein tau (MAPT), forming neurofibrillary tangles (NFT). Cerebral atrophy, microglial activation, oxidative stress, and chronic inflammation of the brain are also seen postmortem (Chen and Mobley, 2019).

AD is most commonly categorized as either late-onset (> 65 years of age) sporadic AD or early-onset (< 65 years of age) familial AD. Sporadic AD is the most common form of AD and is sporadic in nature, with the most widely studied genetic risk factor for sporadic AD being the gene encoding apolipoprotein E (Liu et al., 2013; Mendiola-Precoma et al., 2016). Familial AD is estimated to represent less than 5% of all AD cases and results from the inheritance of an autosomal dominant mutation in the genes encoding amyloid precursor protein (APP), presenilin 1 (PS1), or presenilin 2 (PS2), the latter two being enzymes participating in the processing of APP. Mutations in *APP*, *PS1*, and *PS2* result in the aberrant cleavage of APP into Aβ peptides of 40 residues (Aβ_40_) or of 42 residues (Aβ_42_), which are thought to form toxic Aβ plaques responsible for causing neuronal cell death in AD (Hardy and Higgins, 1992).

Currently available treatments for AD include three acetylcholinesterase inhibitors (donepezil, rivastigmine, and galantamine) and one N-methyl-D-aspartate (NMDA) receptor antagonist (memantine). These treatments have numerous side effects and only provide symptomatic relief to patients in early disease stages without altering disease progression (Wong, 2016). A recent approach in the race for the treatment for AD involves targeting the endocannabinoid system, which is involved in numerous basic functions of the human body (Di Marzo et al., 2004; Benito et al., 2007), and testing constituents of the *cannabis sativa* plant (i.e., phytocannabinoids). Among the group of phytocannabinoids tested for therapeutic interventions, cannabidiol (CBD) is of particular interest. CBD is the main nontoxic (nonhigh producing) phytocannabinoid of *C. sativa* and possesses antioxidant, antiapoptotic, neuroprotective, immunosuppressive, and anti-inflammatory properties. Limited *in vitro* and *in vivo* evidence suggests that CBD may also reduce amyloid and tau pathologies and unlike other cannabinoids does not impair cognition (reviewed in Karl et al. (2017)). These properties suggest that CBD may be suitable for the treatment of neurodegenerative diseases including dementia.

Indeed, CBD has shown potential as a therapeutic for AD in preclinical studies. *In vitro* studies have shown that CBD dose-dependently inhibits tau hyperphosphorylation in Aβ-simulated PC12 cells (Esposito et al., 2006a). Furthermore, CBD can increase cell survival, reduce Aβ-induced lipid peroxidation and reactive oxygen species production (Iuvone et al., 2004), attenuate nitric oxide (Esposito et al., 2006b), and counteract the elevation of APP expression in transfected human neuroblastoma cells, thereby increasing cell survival (Scuderi et al., 2014). *In vivo*, CBD has been found to attenuate Aβ-evoked neuroinflammation in a pharmacological mouse model of AD (Esposito et al., 2007). In addition, 20 mg/kg CBD treatment has been shown to prevent an Aβ-induced learning deficit in the Morris Water Maze and to reduce the Aβ-induced increase in IL-6 (Martín-Moreno et al., 2011). Furthermore, a previous study from our lab reported that CBD at a dose of 20 mg/kg reversed social recognition and novel objection recognition deficits in 6-month-old *APP*_*Swe*_ */PS1ΔE9* (*APPxPS1*) mice (a transgenic model for familial AD) when delivered chronically after the onset of disease-relevant symptoms (Cheng et al., 2014a). This dosage also prevented the development of a social recognition deficit in the *APPxPS1* model when delivered for 8 months prior to the onset of disease symptoms (Cheng et al., 2014c). More recently, 50 mg/kg CBD was found to restore impaired social recognition memory and reversal spatial learning and tended to reduce insoluble Aβ40 levels in the hippocampus of 12-month-old *APPxPS1* males (Watt et al., 2020). International colleagues also evaluated CBD-rich cannabis extract (at a dose of 0.75 mg/kg of CBD) which also improved object recognition memory of *APPxPS1* mice when chronically administered during the early symptomatic disease stage (Aso et al., 2015).

The *APPxPS1* mouse model exhibits fast-developing amyloid pathology (Borchelt et al., 1997; Jankowsky et al., 2004a; Jankowsky et al., 2004b), with Aβ plaques appearing as early as at 4–6 months of age and accumulating further with age (Jankowsky et al., 2004b; Savonenko et al., 2005; Garcia-Alloza et al., 2006; Ruan et al., 2009; Hamilton and Holscher, 2012). A sexual dimorphism profile for this model has also been identified, with female *APPxPS1* mice exhibiting higher pathological levels of phosphorylated tau, proinflammatory cytokines, astrocytosis, microgliosis, neuronal and synaptic degeneration (Jiao et al., 2016), and soluble Aβ40 and Aβ42 peptides (Wang et al., 2003) when compared to males. *APPxPS1* mice also exhibit a range of behavioral deficits relevant to the study of AD including spatial learning and memory impairments in various test paradigms (Savonenko et al., 2005; O’Leary and Brown, 2009; Zhang et al., 2011; Cheng et al., 2014b) and recognition memory impairments (NORT; Lalonde et al., 2004; Cheng et al., 2013; Cheng et al., 2014a; Cheng et al., 2014c) as well as task-dependent hyperlocomotion and anxiolytic-like phenotypes (Cheng et al., 2013; Cheng et al., 2014b). In line with brain pathology, male and female *APPxPS1* mice show differences in the nature of their behavioral impairments (Jardanhazi-Kurutz et al., 2010; Cheng et al., 2014a; Cheng et al., 2014b) so research strategies need to be developed sex-specifically. Unlike previous studies that combined male and female mice and assessed them together without consideration for the impact of gender (Lalonde et al., 2004; Reiserer et al., 2007), the current study decided to limit investigations to one gender only, and female mice were selected because of their more pronounced brain pathology.

It is important to note that CBD produces biphasic dose responses (Tzavara et al., 2003; Rey et al., 2012). It is therefore pivotal to investigate a range of dosages to determine the window of the therapeutic effectiveness of the drug. In addition, evaluating lower CBD doses than in our previous studies may have a positive impact on future financial burdens of dementia patients. Thus, the major aim of this explorative study was to determine if a chronic administration regime of a medium CBD dose of 5 mg/kg bodyweight can reverse or ameliorate behavioral impairments of *APPxPS1* transgenic females at an advanced symptomatic disease stage.

## Materials and Methods

### Animals

12-month-old female double transgenic *APP*_*Swe*_ */PS1ΔE9* (*APPxPS1*) mice were used in this study. The *APPxPS1* mouse model of familial AD carries the chimeric mouse/human *APP* gene with Swedish mutation (Mo/HuAPP695swe/Swedish mutations K595N/M596L) and the mutant human *PS1* gene with exon nine deletion (*PS1/ΔE9*) and is generated on a mixed congenic C57BL/6JxC3H/HeJ background and maintained as a hemizygote (Borchelt et al., 1997; Jankowsky et al., 2004a; Jankowsky et al., 2004b). 12-month old female mice which were chosen as *APPxPS1* females show significantly higher levels of soluble Aβ_40_ and Aβ_42_ compared to male *APPxPS1* mice (Wang et al., 2003) and at 12 months of age, these females are considered to be in advanced stages of the symptomatic phase of AD (Aso et al., 2016). *APPxPS1* mice (*n* = 22) and their nontransgenic wild type-like littermates (WT: *n* = 28) were 361 ± 8 days old at the onset of the study, with a total of three cohorts of mice being used. Mice were bred at Australian BioResources (ABR: Moss Vale, NSW Australia) where they were group housed in individually ventilated cages (Type Mouse Version 1: Airlaw, Smithfield, Australia) under a 12/12 h light/dark cycle with a dawn/dusk simulation. Mice were transported to the Western Sydney University animal facility (School of Medicine, Campbelltown, Australia) once they had reached adulthood where littermates were group housed (two to three mice per cage) in filter top cages (1284L: Tecniplast, Rydalmere, Australia). Mice were provided with food (Rat & Mouse Pellets: Gordon’s Specialty Stockfeeds Pty Ltd., NSW, Australia) and water *ab libitum* unless otherwise described. Corn-cob bedding (PuraCob Premium: Able Scientific, Perth, Australia), crinkle paper (Crink-l’Nest, The Andersons, Maumee, Ohio, United States), and tissue for nesting were used as enriching structures. Cages were changed fortnightly. Standard laboratory conditions were applied with a 12/12 h light/dark cycle (light phase beginning 0900 with white light at an illumination of 124 lux and dark phase beginning 2100 with a red light at an illumination of less than 2 lux). Temperature and relative humidity were automatically controlled between 20 and 22°C and 40 and 60%, respectively. All procedures were approved by the Western Sydney University Animal Care and Ethics Committee (#A12905) and complied with the *Australian Code of Practice for the Care and Use of Animals for Scientific Purposes*.

### Drug Preparation and Administration

Preparation of powdered cannabidiol (CAS: 13956-29-1; THC Pharma GmbH, Frankfurt/Main, Germany) dissolved to a concentration of 0.5 mg/ml in equal parts of Tween80 (Sigma-Aldrich Co., St Louis, United States) and 100% ethanol and diluted in 0.9% saline, to a ratio by volume of 1 : 1 : 18 ethanol: Tween80: saline, was used to prepare the CBD treatment solution. A similar solution without the addition of powdered cannabidiol (1 : 1 : 18 ethanol : Tween80 : saline) was used as the vehicle. At approximately 12 months of age, mice began treatment via daily intraperitoneal (i.p.) injection (10 ml/kg bodyweight, site alternated daily) of CBD or vehicle administered at a dose of 5 mg/kg body weight (WT-VEH *n* = 15; WT-CBD *n* = 13; *APPxPS1*-VEH *n* = 10; and *APPxPS1*-CBD *n* = 12). Treatment began 3 weeks prior to the start of the experiments and continued throughout the behavioral assessment. CBD or vehicle was administered in the afternoon to avoid acute effects of the injections modifying the behavioral performance of the mice tested in the morning, in line with our other studies (Cheng et al., 2014a; Watt et al., 2020). Bodyweight was monitored weekly.

### Behavioral Test Battery

Mice were tested in behavioral domains that have been found to be affected in dementia or AD-relevant mouse models. In line with previous studies conducted in our laboratory (Cheng et al., 2014a; Watt et al., 2020), all experiments were performed during the first 5 h of the light phase to reduce the effects of the circadian rhythm on mice performance (i.e., to avoid the less active period of the light phase (Grech et al., 2019)), and a 48 h intertest interval was applied to all testing to minimize the effect of repeated testing and to allow mice to rest between tests (with the exception of low-impact motor function tests, which were performed over three consecutive days). Mice were habituated to the test room for 30–60 min prior to testing. 80% ethanol was used to clean each apparatus between mice. For an overview of test order and test age, please see Table 1.

**TABLE 1:** Test biography. Test order and test age of wild type-like (WT) control and double transgenic *APP*_*Swe*_ */PS1ΔE9* (*APPxPS1*) female mice treated with either vehicle (VEH) or cannabidiol (CBD). Ages (d) are presented as mean ± standard error of means (SEM). NORT: novel object recognition task. No significant differences between days of test.

	WT-VEH	*APPxPS1*-VEH	WT-CBD	*APPxPS1*-CBD
Start of CBD treatment	361 ± 2	357 ± 2	363 ± 2	360 ± 2
Light-dark test	382 ± 2	378 ± 2	384 ± 2	381 ± 2
Pole test	384 ± 2	380 ± 2	386 ± 2	383 ± 2
Accelerod	384 ± 2	380 ± 2	386 ± 2	383 ± 2
NORT	389 ± 2	385 ± 2	391 ± 2	388 ± 2
Cheeseboard	392 ± 2	388 ± 2	394 ± 2	391 ± 2
Prepulse inhibition	406 ± 2	404 ± 2	408 ± 2	405 ± 2

#### Light Dark

Anxiety-related behaviors can be assessed in the light-dark (LD) test. The LD apparatus (for details, see Karl et al. (2007), Cheng et al. (2014b)) consisted of two equally sized zones in an open-field chamber: a “light” zone (illumination > 200 lux) and a “dark” zone (illumination < 20 lux; dark box insert in the rear half of test arena). After a 60 min habituation to the test room, mice were placed into the dark zone and allowed to explore the entire apparatus for 10 min. The activity was recorded by MED Associates Activity Monitor software. Distance traveled was used as an indicator of locomotion. Exploration was shown by the frequency of *rearing* (vertical activity). Time spent and percentage distance traveled in the light zone were calculated to identify anxiety-related behaviors.

#### Pole Test

Climbing behavior was assessed using the vertical pole test (Brooks and Dunnett, 2009). Mice were placed with snouts facing upwards on the end of a vertical pole (50 cm long by 1 cm diameter) and allowed to turn around and climb down the pole to a platform. This was repeated three times with a 30 min intertrial interval (ITI). The performance was measured by the average time taken to (1) turn around (latency to inversion) and (2) descend the pole once turned around (time to descend) and (3) total time taken to reach the platform (latency to platform; “cut-off” time of 60 s).

#### Accelerod

An accelerating rotarod paradigm was used to measure the motor coordination and balance of the test mice (Brooks and Dunnett, 2009). Training and testing were carried out as described previously (Kreilaus et al., 2019), but with two consecutive test days with two trials per day. The mean of the four trials was considered for analysis, as was the worst-performing trial. The performance was measured as the latency to fall from the cylinder (“cut-off” time of 300 s).

#### Novel Object Recognition Task

The innate preference of a mouse for novelty and its ability to distinguish a novel object from a familiar object (Dere et al., 2007) are utilized in this test to determine object recognition memory. The NORT was conducted as published previously (Kreilaus et al., 2019). The percentage of time spent *nosing* the novel object during the second “testing” trial was calculated as

$$\;\frac{\text{novel object nosing time}}{\text{novel }+\text{ familiar object nosing time}}\;\times 100,\;$$

and it was used as an indication of object recognition memory. In line with previously published studies from our lab (Cheng et al., 2014a), mice were excluded if they did not show a minimum of 20 s of object exploration during both trials (one WT-VEH and one *APPxPS1*-CBD mouse were excluded).

#### Cheeseboard

Spatial memory was assessed through the cheeseboard (CB) paradigm. Details on the apparatus used can be found in previous studies from our lab (Cheng et al., 2013; Kreilaus et al., 2019; Watt et al., 2020). Briefly, mice were habituated over two days to the blank side of the board (i.e., 3 × 2 min trials per day, 20 min ITI). Next, mice were trained over five days to locate a well containing a food reward (i.e., 3 trials per day, 20 min ITI). The latency of the mice to find the baited well was recorded and if the mouse had not found the food reward within the maximum trial time of 2 min, it was gently guided to the well by the experimenter. To ensure motivation to find the food reward (i.e., sweetened condensed milk), mice were food restricted (access to food for 1–2 h following completion of daily testing) to a maximum of 85% of their free-feeding body weight throughout the entire testing period.

The average latency to find the reward and the mean speed and distance traveled during training were analyzed as a general indication of learning, while the first trial per day across training was analyzed to assess long-term reference memory (retention of ≥ 24 h), and the average of trials two and three each day across training was analyzed to assess intermediate-term memory (retention falling between short-term (2 min) and long-term (24 h) memory) (Taglialatela et al., 2009). Further, day-by-day learning in the CB, where the average latency for day 1 was compared to day 2, day 3, and so on, was performed to determine when the mice acquired the task.

A CB probe trial for spatial memory was performed on day 8, whereby mice were given 2 min to explore the board with no food reward present. The percentage of duration spent in the target zone (the zone containing the target well during training, i.e., 12.5% of the board) was analyzed using AnyMaze™ (Stoeting, Wood Dale, United States) tracking software, thereby analyzing target zone preference for total test time. As it has been observed that some mice do not leave the center zone immediately and therefore do not spend the entire 2 min of the probe trial exploring the board, a secondary calculation was carried out to ensure that the data presented was representative of the actual test time that mice spent exploring and was not skewed by an extended latency to leave the central start zone. This was calculated as

$$\frac{\text{time }\left(\text{s}\right)\text{ in target zone}}{120\;s\;-\;\text{latency }\left(\text{s}\right)\text{ to leave the centre zone}}\;\times \;100.$$

The percentage of duration spent in the target zone for the first and second 30 s of the full 2 min trial was also analyzed to account for potential differences in behavioral flexibility rather than spatial memory, as a target zone preference in the first/second 30 s is indicative of intact retrieval memory or perseverative behavior, respectively, while decreased time in the target zone over the second 30 s is indicative of cognitive flexibility in adaptation to the lack of food reward (Grech et al., 2019). A reversal CB was also completed (4 days of training followed by a reversal probe trial, where the opposite well was baited). One *APPxPS1*-CBD mouse was excluded from probe analysis as it *froze* for 80 s (three times greater than any other mouse).

#### Prepulse Inhibition

The prepulse inhibition (PPI) test was used to assess the acoustic startle response (ASR) and sensorimotor gating (the occurrence by which a nonstartling prestimulus attenuates the startle response (Wang et al., 2012)). Mice were habituated to the apparatus (apparatus described in Cheng et al. (2014b)) for 10 min twice per day (1 h ITI) over two consecutive days prior to the test day. On day 3, mice were returned to the apparatus for the PPI test, which was carried out as previously described (Cheng et al., 2014b). A 120 dB startle pulse, prepulse intensities of 74, 82, and 86 dB, and interstimulus intervals of 32, 64, 128, and 256  ms were used in this test protocol. Percentage PPI (%PPI) was calculated as

$$\frac{\text{mean startle response }\left(120\text{ dB}\right)\;-\;\text{PPI response}}{\text{mean startle response }\left(120\text{ dB}\right)}\;\times \;100.$$

%PPI was averaged across ISIs to produce a mean %PPI for each prepulse intensity.

### Statistical Analysis

Analysis of behavioral data was performed using two-way ANOVA to determine the main effects of between-subject factors “genotype” and “treatment” and to test for “genotype” by “treatment” interactions. Three-way repeated measure (RM) ANOVA was also used to investigate repeated measure effects of the within-subject factors “time” (CB), “startle pulse intensity,” “startle block,” and “prepulse intensity” (all PPI). A “time” by “genotype” by “treatment” interaction was further investigated in CB by splitting data by “genotype” and then by both “genotype” and “treatment” and using mixed ANOVA and/or one-way RM ANOVA, respectively. To investigate day-by-day learning in CB, one-way RM ANOVA for “time” for day 1 versus respective day/s was performed. One-sample *t*-tests were also used for NORT and CB probe to determine whether a specific behavior was above chance levels (i.e., 50% for NORT–12.5% for CB). In line with Perneger (1998) and Rothman (1990), the data were not adjusted for multiple comparisons and were interpreted as such in Discussion. Significant differences were determined when *p* < .05. F-values and degrees of freedom are presented for ANOVA and significant effects of “genotype” are shown in figures and tables by “*” (*p < .05, **p < .01, and ***p < .001), and significant effects of “treatment” are shown by “^#^” (^#^ *p* < .05). Significant RM results are indicated by ‘ ^ ’ (^ *p* < .05, ^ ^*p* < .01, and ^ ^ ^*p* < .001). A “time” by “treatment” interaction is indicated by “^†^” (^†^ *p* < .05). Significant *t*-test results are also shown by “^+^” (^+^ *p* < .05, ^++^ *p* < .01, and ^+++^ *p* < .001). Trends were reported when .05 ≤ *p* < .07, and all other nonsignificant data were reported as “n.s.” (i.e., *p* ≥ .07) or with specific *p*-values. Data are shown as means ± standard error of means (SEM). All statistical analyses were conducted using IBM SPSS Statistics 25.0 for Mac.

## Results

### Locomotion and Exploration

*APPxPS1* mice displayed increased total distance traveled in the LD test (two-way ANOVA for “genotype”: F (1,46) = 9.2 and *p* = .004) and this increase in locomotion was not affected by treatment (i.e., no “genotype” by “treatment” interaction: F (1,46) = .07 and *p* = .8, Table 2). Importantly, this hyperlocomotive phenotype of *APPxPS1* mice was also evident in the dark zone, which is least affected by anxiety behaviors (F (1,46) = 16.3 and *p* < .001, Table 2). No differences in exploration (i.e., *rearing* frequency) were detected between genotypes for the LD arena or any particular zone (all *p’s* n.s., Table 2). Interestingly, mice treated with CBD exhibited increased *rearing* compared to vehicle-treated mice in the dark zone (F (1,46) = 5.5 and *p* = .02, Table 2) but no other LD area (all *p’s* n.s.). However, follow-up analysis of exploration per minute spent in this zone did not detect a treatment effect (F (1,46) = 1.1 and *p* = .3, Table 2).

**TABLE 2:** Locomotion, exploration, and anxiety measures in the light-dark test. Data shown for wild type-like (WT) control and double transgenic *APP*_*Swe*_ */PS1ΔE9* (*APPxPS1*) female mice treated chronically with either vehicle (VEH) or cannabidiol (CBD). Data are presented as mean ± SEM. Main “genotype” effects are presented as *p < .05, **p < .01, and ***p < .001 or trend value given. Significant main effects of “treatment” are indicated by “^#^” (^#^ *p* < .05).

	WT-VEH	*APPxPS1-*VEH	WT-CBD	*APPxPS1*-CBD
Total distance traveled (m)**	32.5 ± 1.6	40.0 ± 2.9	32.4 ± 1.5	38.7 ± 3.1
Dark zone distance traveled (m)***	17.0 ± 1.1	21.4 ± 1.8	17.1 ± .9	23.4 ± 1.5
Total *rearing* frequency (n)	120.1 ± 7.8	129.0 ± 15.1	128.7 ± 11.1	141.9 ± 12.7
Dark zone *rearing* frequency (n)^#^	51.3 ± 2.5	56.5 ± 6.7	60.5 ± 7.2	76.0 ± 7.6
Dark zone *rearing* frequency per minute in the zone (n/min)	12.2 ± 1.1	12.3 ± 1.7	13.4 ± 1.4	14.2 ± 1.6
Time spent in light zone (s)*	294.1 ± 17.8	277.6 ± 27.0	290.4 ± 8.4	230.9 ± 15.8
Distance traveled in light zone (%), *p* = .06	47.2 ± 2.4	46.1 ± 3.7	47.2 ± 1.6	38.6 ± 2.4

### Anxiety

*APPxPS1* transgenic mice were more anxious than controls during LD testing. In particular, *APPxPS1* mice spent significantly less time in the light zone of the LD test (F (1,46) = 4.6 and *p* = .04, Table 2) and they also tended to exhibit less locomotion in that zone (i.e., percentage distance traveled: F (1,46) = 3.6 and *p* = .06, Table 2). Chronic CBD had no effect on anxiety parameters or genotype differences detected (all *p’s* n.s.).

### Motor Function

In the pole test, no significant main effects of *APPxPS1* genotype or CBD treatment were found for the measures latency to inversion, time to descend, and latency to platform (all *p’s* n.s., Table 3). Similarly, in the accelerod, no effects of “genotype” or “treatment” were evident for the average latency to fall from the accelerod (all *p*’s n.s., Table 3). However, *APPxPS1* mice fell from the accelerod significantly earlier than controls when comparing the worst performance of test mice across trials (F (1,46) = 7.1 and *p* = .01, Table 3) and this was not affected by CBD (no “genotype” by “treatment” interaction: F (1,46) = .3 and *p* = .6).

**TABLE 3:** Motor functions in the pole test and accelerod. Data shown for wild type-like (WT) control and double transgenic *APP*_*Swe*_ */PS1ΔE9* (*APPxPS1*) female mice treated with either vehicle (VEH) or cannabidiol (CBD). Data are presented as mean ± SEM. Significant “genotype” effects are indicated with “*” (*p < .05).

	WT-VEH	*APPxPS1*-VEH	WT-CBD	*APPxPS1*-CBD
Pole test, latency to inversion (s)	13.8 ± 2.9	14.0 ± 3.8	7.6 ± 1.2	10.7 ± 2.5
Pole test, time to descend (s)	17.8 ± 2.0	18.2 ± 2.7	15.0 ± 1.3	18.0 ± 2.2
Pole test, latency to platform (s)	31.6 ± 4.4	32.2 ± 5.2	22.7 ± 1.8	28.7 ± 3.9
Accelerod, average latency to fall (s)	202.9 ± 9.1	171.3 ± 11.7	193.9 ± 17.1	176.2 ± 17.8
Accelerod, latency to fall in worst performance (s)*	170.8 ± 9.3	120.5 ± 13.8	151.8 ± 17.6	119.0 ± 20.1

### Cognition

#### Object Recognition Memory

**FIGURE 1:**
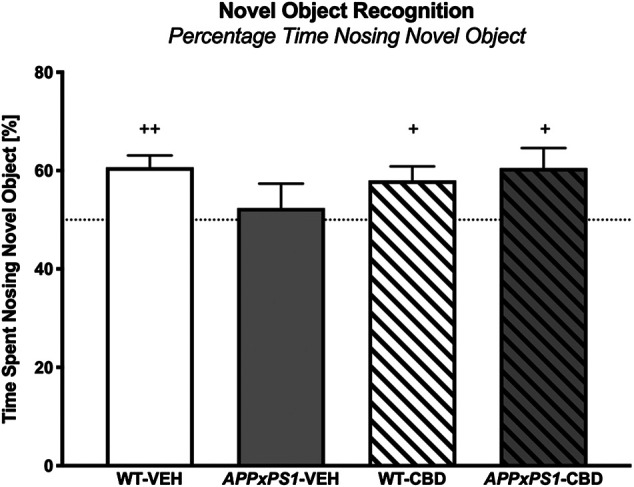
Novel object recognition. The percentage of time spent *nosing* the novel object in the NORT. Data for wild type-like (WT) control and double transgenic *APP*_*Swe*_ */PS1ΔE9* (*APPxPS1*) female mice treated with either vehicle (VEH) or cannabidiol (CBD) are shown as means + SEM. Significant *t*-test results against chance levels (i.e., 50%) are indicated with “^+^” (^+^ *p* < .05 and ^++^ *p* < .01).

In the NORT testing trial, all experimental groups except for vehicle-treated *APPxPS1* transgenic mice had a significant preference for the novel object, as indicated by one-sample *t*-tests for the percentage time spent *nosing* the novel object (WT-VEH: *t* (13) = 4.5 and *p* = .001; *APPxPS1*-VEH: *t* (9) = .5 and *p* = .6; WT-CBD: *t* (12) = 2.8 and *p* = .02; *APPxPS1*-CBD: *t* (9) = 2.6 and *p* = .03, Figure 1). Comparing percentage time spent *nosing* the novel object across experimental groups using 2-way ANOVA did not reveal significant main effects or interaction thereof (all *p’s* n.s.).

#### Cheeseboard - Spatial Learning and Memory

##### Task Acquisition

**FIGURE 2:**
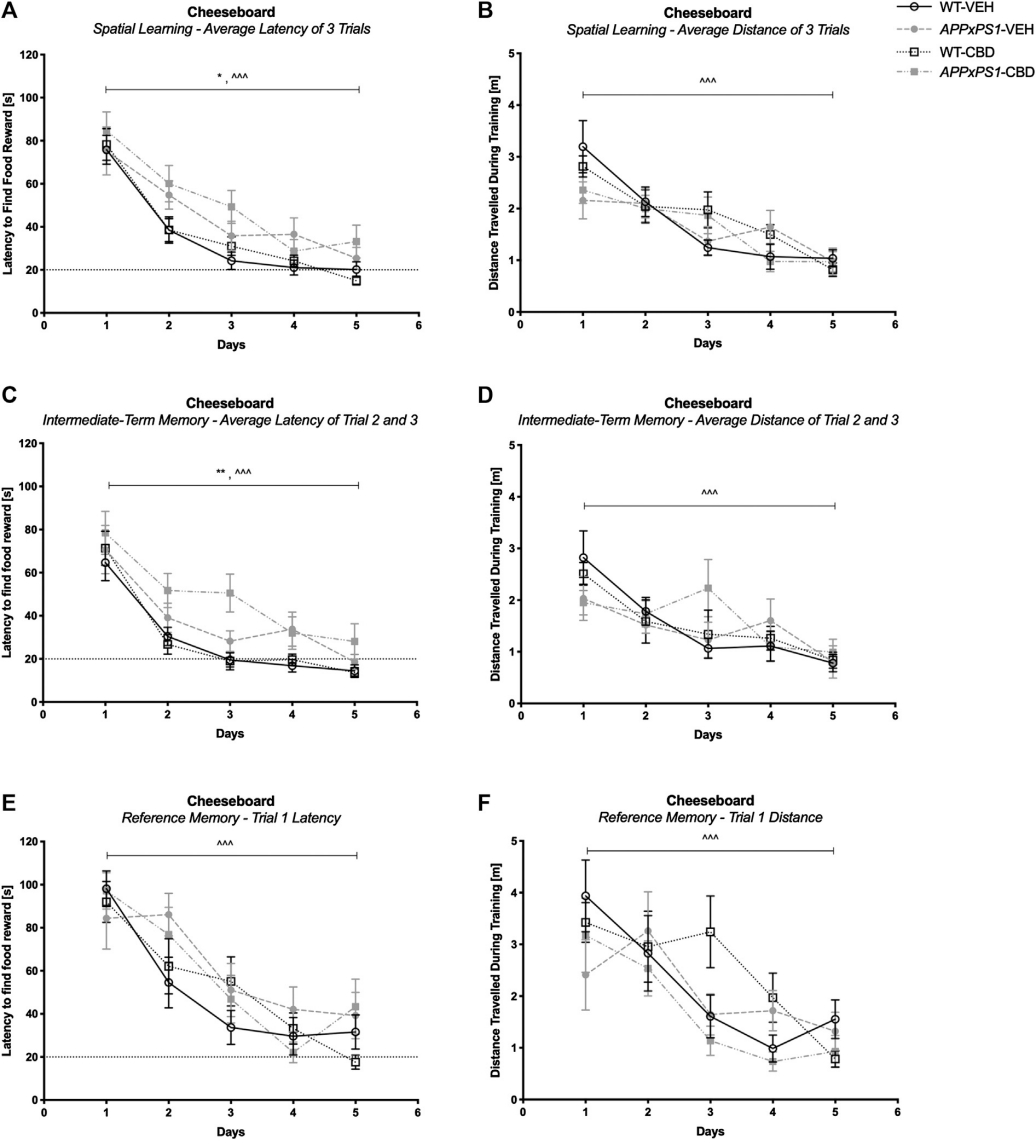
Spatial learning in the cheeseboard (CB). **(A, C, and E)** Latency (s) to find the food reward and **(B, D, and F)** distance traveled (m) during CB training **(A and B)** averaged across all three trials, **(C and D)** for intermediate-term memory and **(E and F)** for reference memory. Data for wild type-like (WT) control and double transgenic *APP*_*Swe*_ */PS1ΔE9* (*APPxPS1*) female mice treated with either vehicle (VEH) or cannabidiol (CBD) are shown as means ± SEM. Significant “genotype” effects are indicated by “*” (*p < .05 and **p < .01) and successful learning is indicated by ‘ ^ ’ (^ ^ ^ *p* < .001).

In the CB training trials, all mice demonstrated successful task acquisition as they learned the position of the baited well. This was indicated by a reduced latency to find the food reward over time and reduced distance traveled during training when averaged across the three trials per day (three-way RM ANOVA for “time”: latency: F (4,180) = 88.6 and *p* < .001; distance: F (4,180) = 23.4 and *p* < .001) and successful learning was not affected by genotype or treatment (no interactions of “genotype” or “treatment” with “time”; all *p*’s n.s., Figures 2A and B). In line with this, intact learning was evident in all groups for both intermediate-term memory (i.e., averaged across trials two and three per day) (latency: F (4,180) = 59.3 and *p* < .001; distance: F (4,180) = 11.6 and *p* < .001, Figures 2C and D) and reference memory (i.e., trial one per day) (latency: F (4,180) = 41.6 and *p* < .001; distance: F (4,180) = 13.1 and *p* < .001, Figures 2E and F) (no interactions of “genotype” or “treatment” with “time”; all *p*’s n.s.). Interestingly, comparing the learning performance of each experimental group separately *day by day* revealed that all groups except the *APPxPS1*-VEH exhibited significant improvement in the latency to find food reward for the first time by day 2 (RM ANOVA for “time” for day 1 *versus* day 2: WT-VEH: F (1,14) = 39.9 and *p* < .001; WT-CBD: F (1,12) = 20.5 and *p* = .001; *APPxPS1*-CBD: F (1,10) = 7.5 and *p* = .02). The learning of the vehicle-treated *APPxPS1* group was delayed and only evident by day 3 (day 1 *versus* day 3, F (1,9) = 10.0 and *p* = .01) (Supplementary Figure S1A).

It should be noted that *APPxPS1* transgenic mice were generally slower on the board than their WT littermates when mean speed was averaged across daily trials, regardless of treatment (F (1,45) = 24.5 and *p* < .001; no interactions with “time” or “treatment,” Supplementary Figure S2A). In line with this, *APPxPS1* mice took generally longer to find the reward than control mice, both averaged across all daily trials (latency to find a reward: F (1,45) = 6.8 and *p* = .01. Figure 2A) and averaged across trials two and three (F (1,45) = 11.1 and *p* = .002, Figure 2C) but no main effect of genotype was detected for reference memory (F (1,45) = 1.3 and *p* = .3, Figure 2E).

##### Reversal Task Acquisition

**FIGURE 3:**
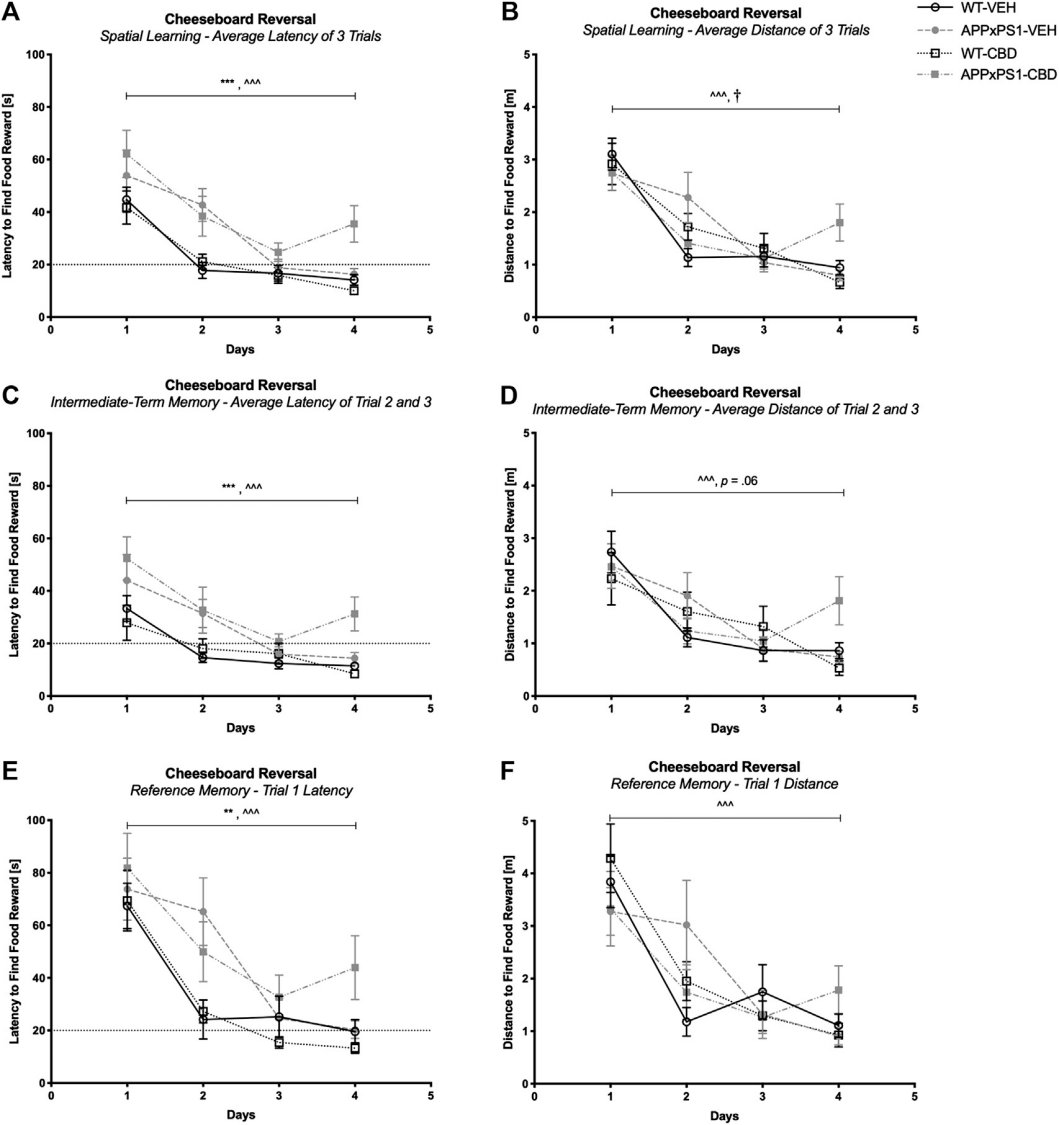
Spatial learning in the reversal cheeseboard (reversal CB). **(A, C, and E)** Latency (s) to find the food reward and **(B, D, and F)** distance traveled (m) during reversal CB training **(A and B)** averaged across all three trials, **(C and D)** for intermediate-term memory and **(E and F)** for reference memory. Data for wild type-like (WT) control and double transgenic *APP*_*Swe*_ */PS1ΔE9* (*APPxPS1*) female mice treated with either vehicle (VEH) or cannabidiol (CBD) are shown as means ± SEM. Significant “genotype” effects are indicated by “*” (**p < .01 and ***p < .001) and successful learning is indicated by ‘ ^ ’ (^ ^ ^ *p* < .001). There was a “time” by “genotype” by “treatment” interaction for distance traveled across all three trials (*p* = .004) and for intermediate-term memory (*p* = .04). The “time” by “treatment” interactions for *APPxPS1* mice are indicated by “^†^” (^†^ *p* < .05) or the exact trend level has been indicated by “*p* = .06.”

Successful reversal training was evident in all mice when averaged across three trials (latency F (3,135) = 47.2 and *p* < .001; distance: F (3,135) = 40.1 and *p* < .001, Figures 3A and B) and also when considering intermediate-term memory (latency: F (3,135) = 20.5 and *p* < .001; distance: F (3,135) = 18.4 and *p* < .001, Figures 3C and D) and reference memory (latency: F (3,135) = 38.9 and *p* < .001; distance: F (3,135) = 24.1 and *p* < .001, Figures 3E and F). No “time” by “genotype” or “time” by “treatment” interactions were detected for latencies (all *p’s* n.s.). However, there were “time” by “genotype” by “treatment” interactions for distance traveled across three daily trials (F (3,135) = 4.6 and *p* = .004, Figure 3B) and when considering intermediate-term memory (F (3,135) = 2.9 and *p* = .04, Figure 3D). Split by genotype, mixed ANOVA revealed a significant “time” by “treatment” interaction for *APPxPS1* mice when considering the distance traveled for the average of the three trials (distance: F (3,57) = 3.5 and *p* = .02, Figure 3B) and a trend interaction when considering the intermediate-term memory (trend: F (3,57) = 2.6 and *p* = .06, Figure 3D). These interactions were not evident in WT mice (all *p*’s n.s.). However, split by “genotype” and “treatment,” all experimental groups displayed intact learning as indicated by significant RM effects of “time” for all groups (WT-VEH: F (3,42) = 23.8 and *p* < .001; *APPxPS1*-VEH: F (3,27) = 11.2 and *p* < .001; WT-CBD: F (3,36) = 12.7 and *p* < .001; *APPxPS1*-CBD: F (3,30) = 5.7 and *p* = .003). Looking at *day-by-day* learning, WT mice regardless of treatment condition displayed significantly improved latencies to find the food reward by day 2 (RM ANOVA for “time” for day 1 *versus* day 2; WT-VEH: F (1,14) = 30.9 and *p* < .001; WT-CBD: F (1,12) = 9.9 and *p* = .008), whereas *APPxPS1* mice of both treatments show improvement by day 3 (day 1 versus day 3; *APPxPS1*-VEH: F (1,9) = 16.9 and *p* = .003; *APPxPS1*-CBD: F (1,10) = 16.0 and *p* = .003) (Supplementary Figure S1B).

*APPxPS1* mice were also slower on the board than their WT littermates during reversal training when averaged across the three trials per day (F (1,45) = 17.2 and *p* < .001, Supplementary Figure S2B). Again, *APPxPS1* mice took longer per day to find the reward when assessing latency across the average of the three trials (F (1,45) = 17.9 and *p* < .001, Figure 3A), across trials 2 and 3 (F (1,45) = 22.3 and *p* < .001, Figure 3C), and also for trial 1 only (F (1,45) = 7.0 and *p* = .007, Figure 3E). We also detected a “time” by “genotype” by “treatment” interaction (F (3,135) = 3.6 and *p* = .02, Supplementary Figure S2B). Split by genotype, a “time” by “treatment” interaction was evident in WT mice (F (3,78) = 2.8 and *p* = .048) with CBD-treated controls showing a more pronounced increase in average speed across days than the respective vehicle treatment group (*p* n.s. for *APPxPS1* mice).

##### Probe Trial

**FIGURE 4:**
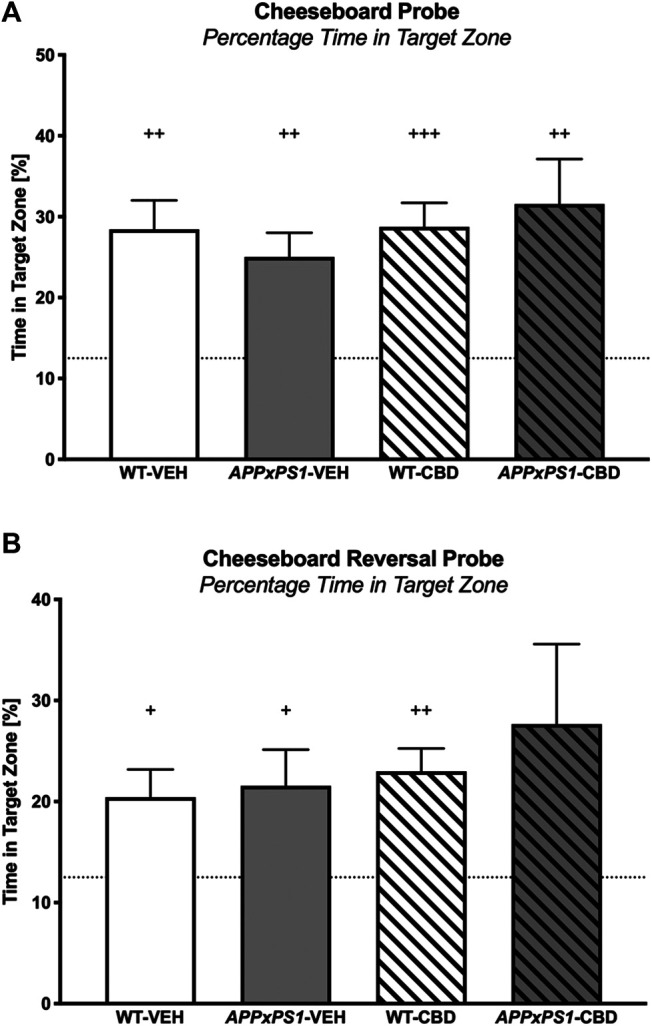
Spatial memory in the cheeseboard (CB) probe and reversal cheeseboard (reversal CB) probe. **(A)** Percentage of time spent (%) in the target zone for the CB probe and **(B)** for the reversal CB probe. Data for wild type-like (WT) control and double transgenic *APP*_*Swe*_ */PS1ΔE9* (*APPxPS1*) female mice treated with either vehicle (VEH) or cannabidiol (CBD) are shown as means + SEM. Significant *t*-test results against chance levels (i.e., 12.5%) are indicated by “^+^” (^+^ *p* < .05,^++^ *p* < .01, and ^+++^ *p* < .001).

During the CB probe trial, all mice showed a preference for the target zone in the full 2 min test period (one-sample *t*-test: WT-VEH: *t* (14) = 4.4 and *p* = .001; *APPxPS1*-VEH: *t* (9) = 4.2 and *p* = .002; WT-CBD: *t* (12) = 5.5 and *p* < .001; *APPxPS1*-CBD: *t* (9) = 3.4 and *p* = .007, Figure 4A). This was also evident when considering the target zone preference postleaving the start zone (WT-VEH: *t* (14) = 4.0 and *p* = .001; *APPxPS1*-VEH: *t* (9) = 4.1 and *p* = .003; WT-CBD: *t* (12) = 5.5 and *p* < .001; *APPxPS1*-CBD: *t* (9) = 3.3 and *p* = .009, Supplementary Figure S3A). Importantly, splitting up the full 2 min probe trial data into 30 s bins, all mice also demonstrated intact retrieval memory in the first bin (WT-VEH: *t* (14) = 4.8 and *p* < .001; *APPxPS1*-VEH: *t* (9) = 3.1 and *p* = .01; WT-CBD: *t* (12) = 5.3 and *p* < .001; *APPxPS1*-CBD: *t* (9) = 3.3 and *p* = .009, Table 4). However, when investigating perseverance in the second 30 s bin, WT mice persevered to find the food reward (WT-VEH: *t* (14) = 3.3 and *p* = .005; WT-CBD: *t* (12) = 3.3 and *p* = .006), whereas *APPxPS1* mice did not (*APPxPS1*-VEH: *t* (9) = 1.8 and *p* = .1; *APPxPS1*-CBD: *t* (9) = 1.2 and *p* = .3, Table 4). Comparing percentage time spent in target zone in the full 2 min test period using two-way ANOVA did not reveal significant main effects or interaction thereof (all *p’s* n.s.).

**TABLE 4:** Retrieval memory and perseverance in the cheeseboard (CB) probe and reversal cheeseboard (reversal CB) probe trials. Data shown as the percentage of time spent in the target zone (%) in the first (indicative of retrieval memory) and second 30 s (indicative of perseverance) of each probe test for wild type-like (WT) control and double transgenic *APP*_*Swe*_ */PS1ΔE9* (*APPxPS1*) female mice treated with either vehicle (VEH) or cannabidiol (CBD). Data are presented as mean ± SEM. Significant *t*-test results against chance levels (i.e., 12.5%) are shown by “^+^” (^+^ *p* < .05, ^++^ *p* < .01, and ^+++^ *p* < .001).

	WT-VEH	*APPxPS1-*VEH	WT-CBD	*APPxPS1*-CBD
*CB probe, % time spent in target zone*				
First 30 s bin	27.5 ± 3.1^+++^	30.3 ± 5.8^+^	34.8 ± 4.2^+++^	34.2 ± 6.5^++^
Second 30 s bin	31.8 ± 5.9^++^	28.9 ± 8.9	22.6 ± 3.0^++^	21.0 ± 7.1
*Reversal CB probe, % time spent in target zone*				
First 30 s bin	29.5 ± 6.0^+^	27.1 ± 4.3^+^	30.2 ± 4.1^++^	34.0 ± 8.0^+^
Second 30 s bin	26.3 ± 3.8^+^	19.1 ± 3.8	21.6 ± 3.2^+^	27.2 ± 8.7

##### Reversal Probe Trial

In the reversal probe trial, all experimental groups (except the *APPxPS1*-CBD group, which was affected by a statistical outlier) had a significant preference for the target zone (WT-VEH: *t* (14) = 2.9 and *p* = .01; *APPxPS1*-VEH: *t* (9) = 2.5 and *p* = .03; WT-CBD: *t* (12) = 4.6 and *p* = .001; *APPxPS1*-CBD: *t* (10) = 1.9 and *p* = .09, Figure 4B) and that preference was also evident when taking into consideration the latency of mice to leave the start zone (WT-VEH: *t* (14) = 2.9 and *p* = .01; *APPxPS1*-VEH: *t* (9) = 2.6 and *p* = .03; WT-CBD: *t* (12) = 4.7 and *p* = .001; *APPxPS1*-CBD: *t* (10) = 2.0 and *p* = .08, Supplementary Figure S3B). As before, all mice demonstrated intact retrieval memory (WT-VEH: *t* (14) = 2.8 and *p* < .01; *APPxPS1*-VEH: *t* (8) = 3.4 and *p* = .01; WT-CBD: *t* (12) = 4.3 and *p* = .001; *APPxPS1*-CBD: *t* (9) = 2.7 and *p* = .03) but *APPxPS1* transgenic mice did not persevere to find the food reward (WT-VEH: *t* (14) = 3.7 and *p* = .002; WT-CBD: *t* (12) = 2.8 and *p* = .02; *APPxPS1*-VEH: *t* (8) = 1.8 and *p* = .1; *APPxPS1*-CBD: *t* (9) = 1.7 and *p* = .1, Table 4). Comparing percentage time spent in target zone in the full 2 min test period using two-way ANOVAs did not reveal significant main effects or interaction thereof (all *p’s* n.s.).

##### Sensorimotor Gating

**FIGURE 5:**
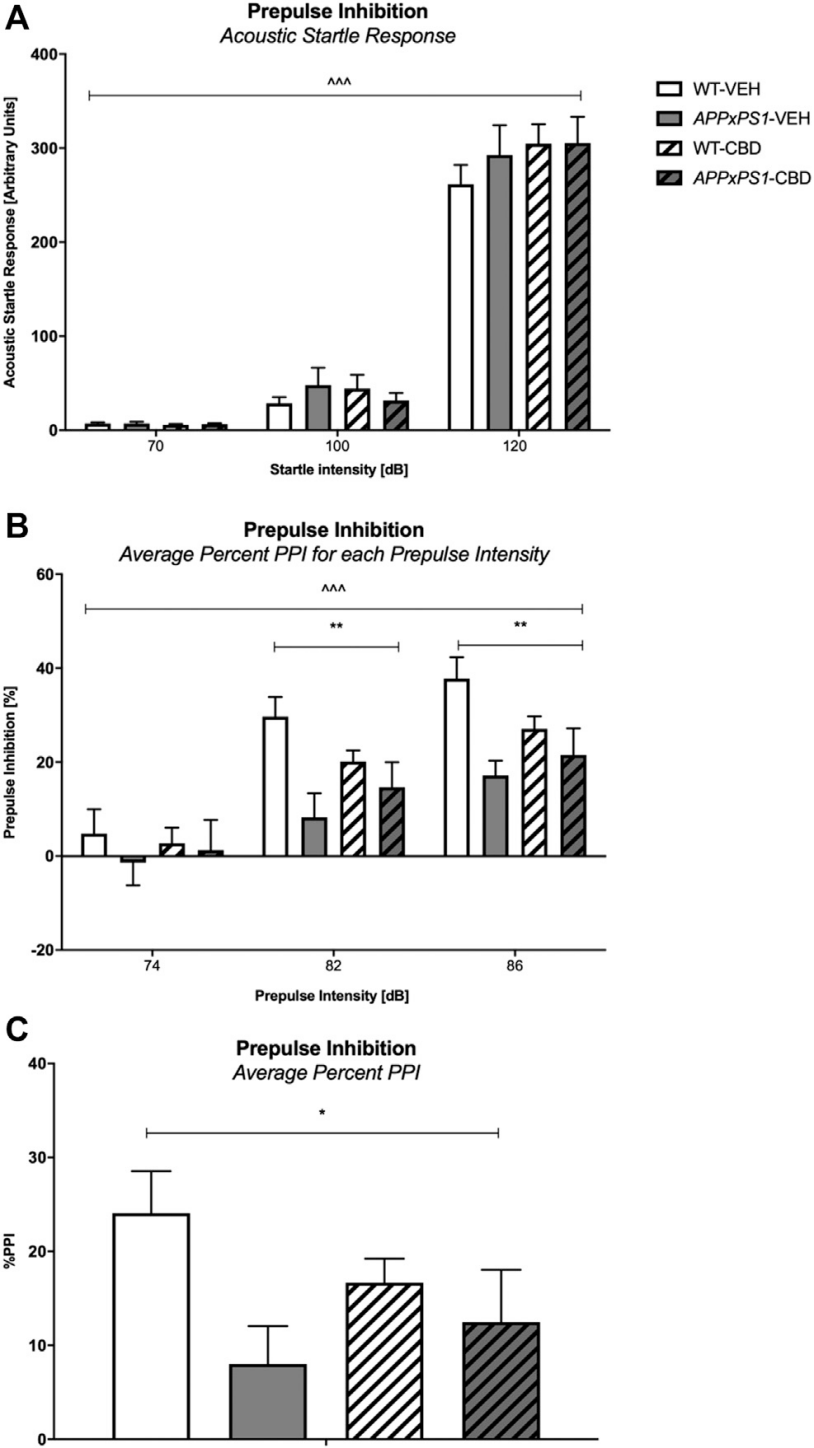
Acoustic startle response (ASR) and sensorimotor gating (PPI). **(A)** ASR to increasing startle pulse intensity (70/100/120 dB), **(B)** percentage prepulse inhibition (%PPI) averaged over trials for increasing prepulse intensities (74/82/86 dB), and **(C)** %PPI averaged over prepulse intensity and interstimulus interval (ISI). Data for wild type-like (WT) control and double transgenic *APP*_*Swe*_ */PS1ΔE9* (*APPxPS1*) female mice treated with either vehicle (VEH) or cannabidiol (CBD) are shown as means + SEM. Significant “genotype” effects are indicated with “*” (*p < .05 and **p < .01) and RM effects are indicated by ‘ ^ ’ ( ^ ^ ^ *p* < .001). There was a “genotype” by “prepulse intensity” interaction for average %PPI for increasing prepulse intensities (*p* < .001).

*Acoustic Startle Response.* The ASR of all mice was similar as there were no main effects of “genotype” or “treatment” (all *p*’s n.s.). Importantly, all experimental groups responded to increasing startle pulse intensities with more pronounced startle responses (RM ANOVA for “startle intensity”: F (2,90) = 411.2 and *p* < .001; no interactions with “genotype” or “treatment”, all *p’s* n.s., Figure 5A). In addition, all mice regardless of test condition displayed decreasing ASR across the three blocks of five 120 dB pulses each, confirming that all mice habituated to the 120 dB startle pulse (RM ANOVA for “startle block”: F (2,90) = 25.4 and *p* < .001; no interactions with “genotype” or “treatment”, all *p’s* n.s., Supplementary Figure S4).

###### Prepulse Inhibition

Three-way RM ANOVA found that as prepulse intensities increased, the %PPI (averaged across ISI) of all mice became more robust as well (“prepulse intensity”: F (2,90) = 166.7 and *p* < .001, Figure 5B). Importantly, a significant “genotype” by “prepulse intensity” interaction (F (2,90) = 8.3 and *p* < .001) was found. Data split by “prepulse intensity” revealed significant effects of “genotype” for %PPI at prepulse intensities of 82 dB (F (1,45) = 9.8 and *p* =.003) and 86 dB (F (1,45) = 9.3 and *p* =.004) but not 74 dB (F (1,45) = .6 and *p* = .5) with *APPxPS1* mice showing reduced prepulse inhibition compared to WT mice (Figure 5B). In line with this, we detected a genotype difference for the average %PPI (F (1,45) = 5.5 and *p* = .02, Figure 5C) with AD transgenic mice exhibiting lower %PPI. CBD treatment had no overall effect on sensorimotor gating and also did not change any genotype effect (i.e., no overall “treatment” effect and no “genotype” by “treatment” interactions for any prepulse intensity; all *p*’s n.s.).

## Discussion

This study demonstrated that chronic administration of a medium dose of 5 mg/kg CBD reversed novel object recognition deficits in 12-month-old female double transgenic *APPxPS1* mice. CBD treatment did not affect the hyperlocomotive or anxiety-like phenotype of the *APPxPS1* mice, nor did CBD moderate the mild motor impairment shown by *APPxPS1* mice. *APPxPS1* mice, although being slower than WT mice, showed intact spatial learning and memory but exhibited impaired perseverance in the CB probe and reversal CB probe, which was not affected by chronic medium-dose CBD. Spatial learning and reversal learning was in fact delayed in *APPxPS1* mice by one day when considering performance across three daily trials compared to WT mice on a day-to-day basis Finally, the ASR of all mice was similar but *APPxPS1* transgenic mice showed a deficit in PPI.

The current study detected an object recognition deficit in 12-month-old *APPxPS1* female mice which is in line with our previous studies in male *APPxPS1* mice tested at the age of 5–6 months (Cheng et al., 2014a) and 12 months (Watt et al., 2020). Furthermore, object recognition impairments in female *APPxPS1* mice have been reported at 12 months of age by international colleagues when using a slightly different test protocol (Aso et al., 2016). Object recognition impairments correlate with the symptomatic stage of the disease, whereby AD patients often have difficulties recognizing faces and objects (Laatu et al., 2003). Importantly, chronic treatment with 5 mg/kg CBD was able to rescue this object recognition deficit. This finding expands on our earlier study reporting therapeutic effectiveness of 20 mg/kg CBD to restore object recognition memory in 5-6-month-old males (Cheng et al., 2014a) and has a similar effect to that seen by Aso et al. (2015) and Aso et al. (2016), whereby a botanical extract containing a combination of delta-9-tetrahydrocannabinol and CBD restored object recognition memory in a V-maze NORT paradigm in 6- and 12-month-old male *APPxPS1* mice, respectively. Interestingly, 50 mg/kg of purified CBD alone did not restore object recognition memory in 12-month-old males (Watt et al., 2020), and this outlines the importance to consider not only dose effects but also testing both male and female mice at early as well as later disease stages. Furthermore, these studies suggest that a combination of cannabinoids may be therapeutically more beneficial (in particular at later disease stages) than CBD alone treatment strategies. Interestingly, impairments in object recognition have been linked to glutamatergic dysfunction and inhibition of the glutamate transporter 1 (Tian et al., 2019), and preclinical studies suggest that antagonism of the glutamate NMDA receptor via memantine can improve object recognition memory (Scholtzova et al., 2008). Importantly, CBD has previously been found to indirectly interact with the NMDA receptor via augmentation of the psychopathological effects of the NMDA receptor antagonist ketamine (Hallak et al., 2011). Thus, CBD may have reversed the object recognition deficits of *APPxPS1* mice in the current study through manipulations of the glutamatergic system. The potential involvement of the glutamate signaling pathway in CBD’s therapeutic-like properties requires further study. The experimental outcomes suggest that lower doses of CBD may have more potential as a therapeutic in clinical settings and at later disease stages and adds further evidence to the biphasic nature of CBD.

The task-dependent hyperlocomotive phenotype of *APPxPS1* female mice confirms and expands our previous findings on task-specific hyperlocomotion in younger AD transgenic mice of both sexes (Cheng et al., 2013; Cheng et al., 2014b). A previous study suggested that this increase in locomotion may be related to increased anxiety or impaired habituation evident in this mouse model (Hooijmans et al., 2009); however, it is important to note that hyperlocomotion and anxiety phenotypes in the *APPxPS1* model appear to be task-specific and are not consistently reported in the literature (see, e.g., O’Leary et al. (2018)). CBD had no effect on the locomotion of WT mice nor on the hyperlocomotive phenotype of *APPxPS1* mice in line with other studies evaluating the effect of various CBD dosing on the locomotion of wild type-like mice (Moreira and Guimarães, 2005; Long et al., 2010; Todd and Arnold, 2016). CBD treatment increased the frequency of *rearing* of both WT and AD transgenic mice specifically in the dark zone, but when corrected for by time, it became clear that CBD had no effect on explorative behavior, confirming previous findings of our laboratory on the absence of CBD effects on *rearing* in male C57BL/6JArc mice (Long et al., 2010).

12-month-old *APPxPS1* female mice displayed an anxiogenic phenotype in the LD test, whereas an anxiolytic-like phenotype was evident in younger, 7-month-old AD females (Cheng et al., 2014b). It is important to note that the *APPxPS1* model of AD shows progressive age-related changes in behavior, cognition, and pathology (Arendash et al., 2001; Trinchese et al., 2004; Pugh et al., 2007; Lok et al., 2013) suggesting a potentially age-dependent change in anxiety behaviors in this mouse model although task and protocol sensitivity of this phenotype have also been raised as potential anxiety behavior-modulating factors in this model (as reviewed in O’Leary et al. (2018)). Importantly, the interpretation of the findings in the LD paradigm is affected by the observation that vehicle-treated WT mice did not show a strong aversion to the light zone. Chronic medium-dose CBD had no effect on anxiety parameters in the LD test, similar to our previous study on the effects of 20 mg/kg CBD in *APPxPS1* males (Cheng et al., 2014a) as well as male and female C57BL/6J mice (although in that study, CBD decreased EPM anxiety (Schleicher et al., 2019)). In this context, it is important to note that CBD has a biphasic dose-response in relation to anxiety effects (Rey et al., 2012; Zuardi et al., 2017). Furthermore, it has been suggested that the anxiolytic effects of CBD may only be evident after an external stressor has been applied, for example, following daily unpredictable stress (Campos et al., 2013).

Motor function impairment has recently been considered as an associated noncognitive symptom of AD (Buchman and Bennett, 2011). In our study, all mice performed equally well in the pole test and in the accelerod, when assessing motor functions across trials. However, *APPxPS1* mice fell off the accelerod sooner than WT mice on their worst-performing trial. Similarly, 6-month-old male and female *APPxPS1* mice tended to slip more often than WT mice in the balance beam test (Kuwabara et al., 2014). Other researches confirm that the motor phenotype of the *APPxPS1* mouse model is task-specific and likely affected by age also (Lalonde et al., 2004; Kemppainen et al., 2014; Kuwabara et al., 2014). Chronic CBD had no impact on motor performance. In line with this, CBD has previously been found to demonstrate few extrapyramidal side effects (Iffland and Grotenhermen, 2017) and not affect motor performance in male Swiss mice either (Ten Ham and De Jong, 1975).

Spatial disorientation is commonly seen in patients with AD (Lithfous et al., 2013). The current study found that the overall ability to acquire the CB and reversal CB task (i.e., the ability to learn the position of a food reward within the overall training period) was not affected in 12-month-old *APPxPS1* mice and that CBD did not affect spatial learning when considering intermediate-term and reference memory. Investigating CB learning in that detail has only recently been described (Kreilaus et al., 2019); thus, this is the first study to identify that the intermediate-term and long-term retention learning memory of 12-month-old *APPxPS1* female mice appear intact. Interestingly, spatial learning and reversal spatial learning of the *APPxPS1* mice were delayed by one day when considering performance across three daily trials compared to WT mice on a day-to-day basis. Importantly, CBD was able to restore this learning delay in the initial training period but did not restore the delay seen in the reversal CB training. It should be noted here that the average speed of *APPxPS1* mice was reduced and therefore transgenic mice took generally longer per day to find the food reward. Chronic CBD had no effect on the spatial learning of control mice in line with previous studies (Fagherazzi et al., 2012).

All mice showed a preference for the target zone indicating intact spatial memory (as well as reversal memory). The target zone preference of *APPxPS1*-CBD mice during reversal testing did not reach significance. However, this appears to be driven by one individual animal, which met the criterion as a statistical outlier but was not excluded from analysis as the mouse did not show any health issues when the test video was reviewed. More stressful tests for spatial memory, that is, the Morris Water Maze, detected memory deficits in 12-month-old and 18-month-old female *APPxPS1* mice (Savonenko et al., 2005; Zhang et al., 2011). Furthermore, 16-month-old *APPxPS1* mice exhibited impaired learning and memory in the Barnes maze (O’Leary and Brown, 2009) suggesting that stress levels may affect the cognitive performance of this AD transgenic mouse model. In addition, our previous work using CB testing detected general spatial memory deficits in 8-9-month-old *APPxPS1* female mice in the reversal CB probe when tested at baseline (Cheng et al., 2014b). Importantly, baseline studies cannot easily be compared to cannabinoid treatment studies as all mice of the latter studies are exposed to daily injections and the necessary handling stress (Gouveia and Hurst, 2019), as well as the effects of the vehicle compound which can shift behavioral phenotypes (Long et al., 2013). Furthermore, the current study was carried out in a new test facility and by a female researcher; both factors have been found to impact on behavioral test outcomes (Lewejohann et al., 2006; Sorge et al., 2014). Finally, we detected intact retrieval memory across experimental conditions but also reduced the persistence of AD transgenic mice to find the food reward. The lack of preference for the target zone of these mice in the second 30 s bin could be discussed as heightened cognitive flexibility in adaptation to the lack of food reward (since the probe trial can be considered as an extinction trial (Grech et al., 2019)). Further analysis into the search patterns of mice during the CB and rCB could be conducted in future studies to determine any deficits in allocentric or egocentric navigational strategies that might explain the lack of perseverance in *APPxPS1* mice during probe trials.

The present study found that the sensorimotor gating of *APPxPS1* mice was reduced compared to control littermates, particularly at higher prepulse intensities, and was not accompanied by any changes in the baseline startle response or habituation thereof. These findings are in line with Wang et al. (2012) who found robust PPI deficits in female mice of a similar *APPxPS1* model as early as at 7 months of age but are different to the work by Cheng and coworkers (Cheng et al., 2014b), which found PPI deficits at the 128 ms ISI only in 10-11-month-old *APPxPS1* female mice (although *APPxPS1* mice in that study “generally” exhibited lower %PPI than WT mice). Chronic treatment with medium-dose CBD did not reverse deficits in PPI. Acute CBD has been found to either attenuate pharmacologically induced disruptions of PPI (but no effect in untreated control mice) (Long et al., 2006; Pedrazzi et al., 2015) or not affect such deficits (Gururajan et al., 2011) and one study utilizing chronic CBD treatment even caused a PPI deficit (Schleicher et al., 2019). Some of these discrepancies may be due to the fact that PPI test outcomes are heavily dependent on the protocol characteristics used in each study (Karl et al., 2011). For example, the experiments of this treatment study compared to the findings of the baseline study of Cheng et al. (2014b) were performed in different locations and utilized different PPI test enclosure habituation procedures (the Cheng protocol used three days of 5 min habituations). It has previously been shown that test location (Karl et al., 2011) and habituation procedures (Swerdlow et al., 2000) are important factors that can impact PPI test outcomes. Furthermore, CBD therapeutic effects on PPI impairments have previously been evaluated in genetic and pharmacological mouse models for schizophrenia, which are characterized by PPI-relevant pathological changes in, for example, the dopaminergic and glutamatergic pathways not necessarily evident in AD mouse models.

The study’s outcome is affected by some limitations: the present study did not investigate male mice for reasons outlined earlier and thus requires follow-up experiments testing the effects of medium-dose CBD on behavioral deficits of *APPxPS1* males. Furthermore, the present study focused on the assessment of the behavioral effects of CBD in this model. Future investigations of AD-relevant neuropathological markers could help explain potential mechanisms regarding the behavioral effects of CBD seen in this study. In brief, *in vitro* studies have shown that CBD acts against Aβ-induced toxicity in various ways, including inhibition of tau hyperphosphorylation (Esposito et al., 2006a) which was associated with a reduction in the phosphorylated glycogen synthase kinase 3-β, the protein responsible for NFT formation in AD. In addition, CBD can increase cell survival, reduce Aβ-induced lipid peroxidation, reactive oxygen species production (Iuvone et al., 2004), and attenuate nitric oxide production via inhibition of phosphorylated p38 mitogen-activated protein kinase and transcription factor nuclear factor-κB (Esposito et al., 2006b). Finally, CBD can counteract the elevation of APP expression by inducing ubiquitination of APP through activation of peroxisome proliferator-activated receptor-γ (PPARγ; Scuderi et al., 2014). In pharmacological mouse models of AD, CBD prevented Aβ-induced spatial learning deficits, reduced the Aβ-induced increase in IL-6 (Martín-Moreno et al., 2011), and attenuated Aβ-evoked neuroinflammation (Esposito et al., 2007) and this appeared to be mediated via PPARγ as well (Esposito et al., 2011). Interestingly, PPARγ has been shown to be elevated in AD patients (de la Monte and Wands, 2006) although our recent research using a 50 mg/kg CBD dose did not find any genotype- or treatment-related changes in this receptor (Watt et al., 2020).

In conclusion, this study found that 12-month-old female *APPxPS1* transgenic mice were hyperlocomotive and showed cognitive impairments (i.e., object recognition memory and spatial learning) as well as PPI deficits. Importantly, chronic treatment with 5 mg/kg CBD reversed object recognition deficits in *APPxPS1* transgenic female mice suggesting a therapeutic-like effect in this established mouse model for AD. To conclude, this study suggests that CBD has therapeutic value for specific behavioral impairments present in AD. Importantly, to date, there is a lack of completed clinical trials on the therapeutic effects of CBD or CBD-rich cannabis extracts on AD symptoms. The current study assists in defining therapeutic dose regimes potentially effective in AD patients and lower-dose CBD treatment would reduce not only the therapy costs for patients but also potential side effects (most important for therapeutic cannabis compounds containing not only CBD but also other cannabinoids such as Δ^9^-tetrahydrocannabinol, as would be the case for CBD-enriched cannabis extract therapies).

## Data Availability Statement

The raw data supporting the conclusions of this article will be made available by the authors, without undue reservation.

## Ethics Statement

The animal study was reviewed and approved by the Animal Care and Ethics Committee of Western Sydney University.

## Author Contributions

TK conceptualized the study design and experimental protocol. MC carried out the drug administration and behavioral experiments following the training provided by GW and FK. MC collected the data and prepared the figures, and TK, MC, GW, and FK performed the statistical analysis. MC and TK drafted and revised the manuscript. All authors approved the submitted version.

## Conflict of Interest

The authors declare that the research was conducted in the absence of any commercial or financial relationships that could be construed as a potential conflict of interest.
