# Supplementary material for: Medium-Dose Chronic Cannabidiol Treatment Reverses Object Recognition Memory Deficits of APP Swe /PS1ΔE9 Transgenic Female Mice
Source: Front Pharmacol. 2020 Dec 4;11:587604. doi: 10.3389/fphar.2020.587604 (PMC7789874; doi:10.3389/fphar.2020.587604)

Supplementary Figure S1A-B: Average latency [s] to find the food reward during CB training **(A)** and reversal CB training **(B)** with *day-by-day* analysis of successful learning for each experimental group. Data for wild type-like (WT) control and double transgenic *APPSwe/PS1ΔE9* (*APPxPS1*) female mice treated with either vehicle (VEH) or cannabidiol (CBD) are shown as means ± SEM. Significant one-way RM ANOVA for ‘time’ for day 1 *versus* respective day on which successful learning was evident indicated by a bracket labelled with the experimental group with respective significance value indicated by ‘^’ ($^{\wedge}p < .05$, $^{\wedge\wedge}p < .01$, and $^{\wedge\wedge\wedge}p < .001$).

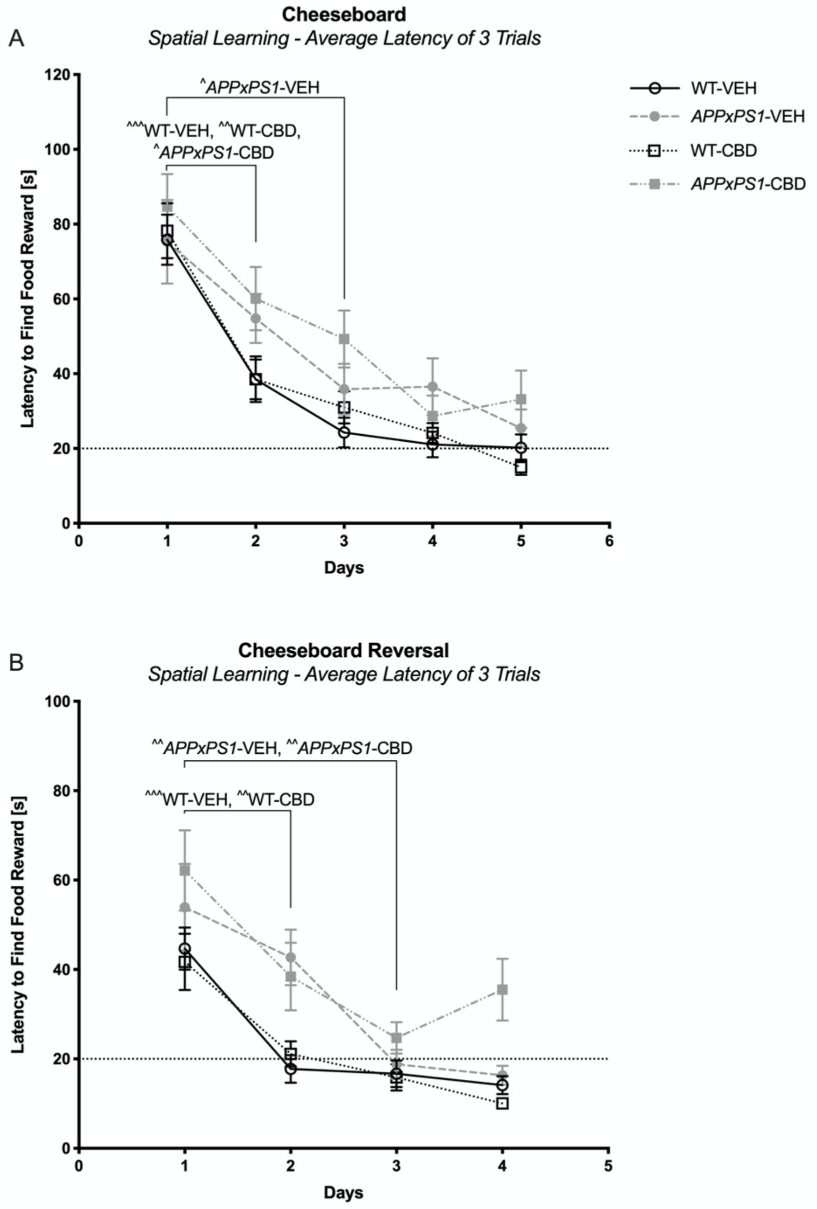

Supplementary Figure S2A-B: Mean speed [m/s] in the cheeseboard averaged across all three trials for **(A)** initial training and **(B)** reversal training. Data for non-transgenic wild type-like (WT) control and double transgenic *APP$_{Swe}$/PS1ΔE9* (*APPxPS1*) female mice treated with either vehicle (VEH) or cannabidiol (CBD) are shown as means ± SEM. Significant 'genotype' main effects are indicated by '*' (****p* < .001). A significant 'time' by 'genotype' by 'treatment' interaction was evident during reversal training (*p* < .001).

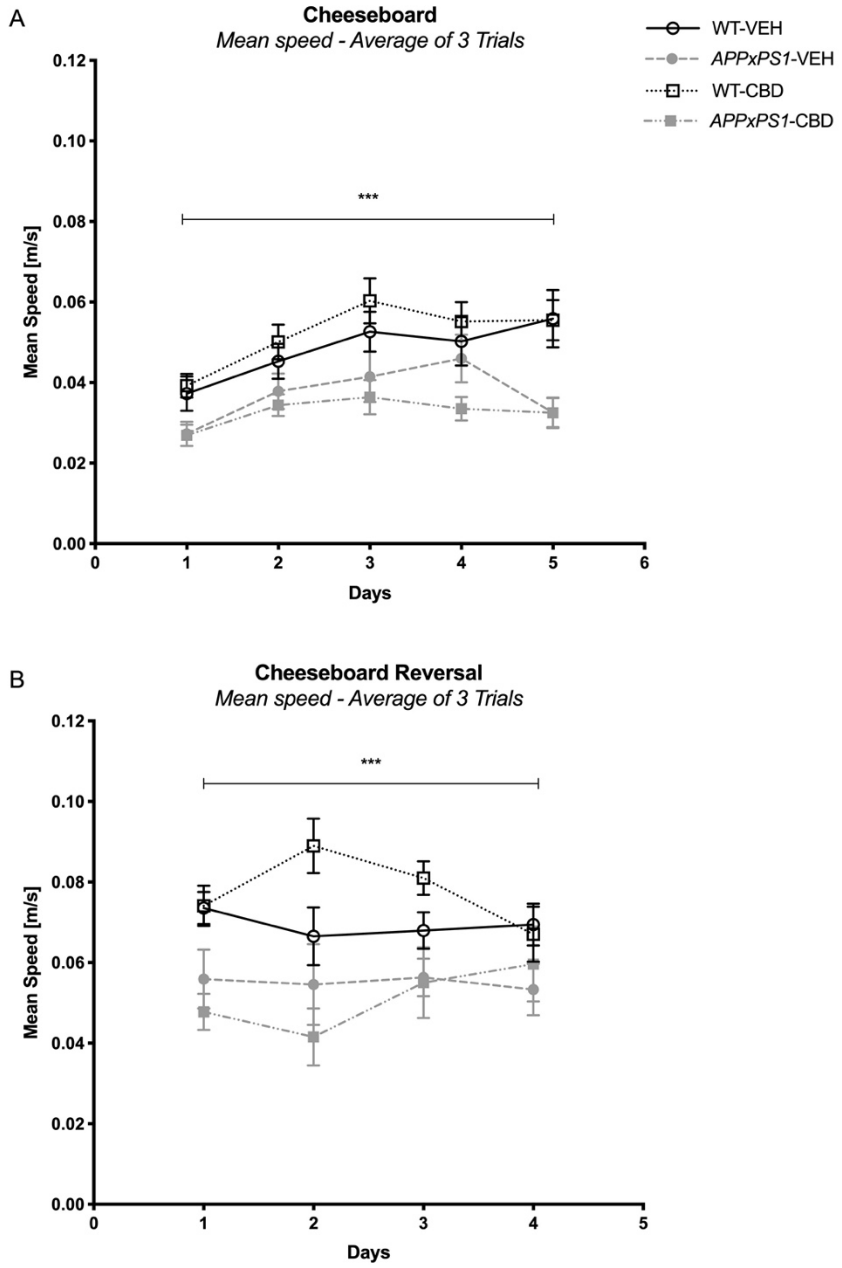

Supplementary Figure S3A-B: Percentage of time spent [%] in the target zone excluding latency to leave the centre zone for **(A)** the CB probe trial and **(B)** the reversal CB probe trial. Data for wild type-like (WT) control and double transgenic *$APP_{Swe}/PS1\Delta E9$* (*APPxPS1*) female mice treated with either vehicle (VEH) or cannabidiol (CBD) are shown as means + SEM. Significant t-test results against chance levels (i.e. 12.5%) are indicated by '+' ($^{+}p < .05$, $^{++}p < .01$ and $^{+++}p < .001$).

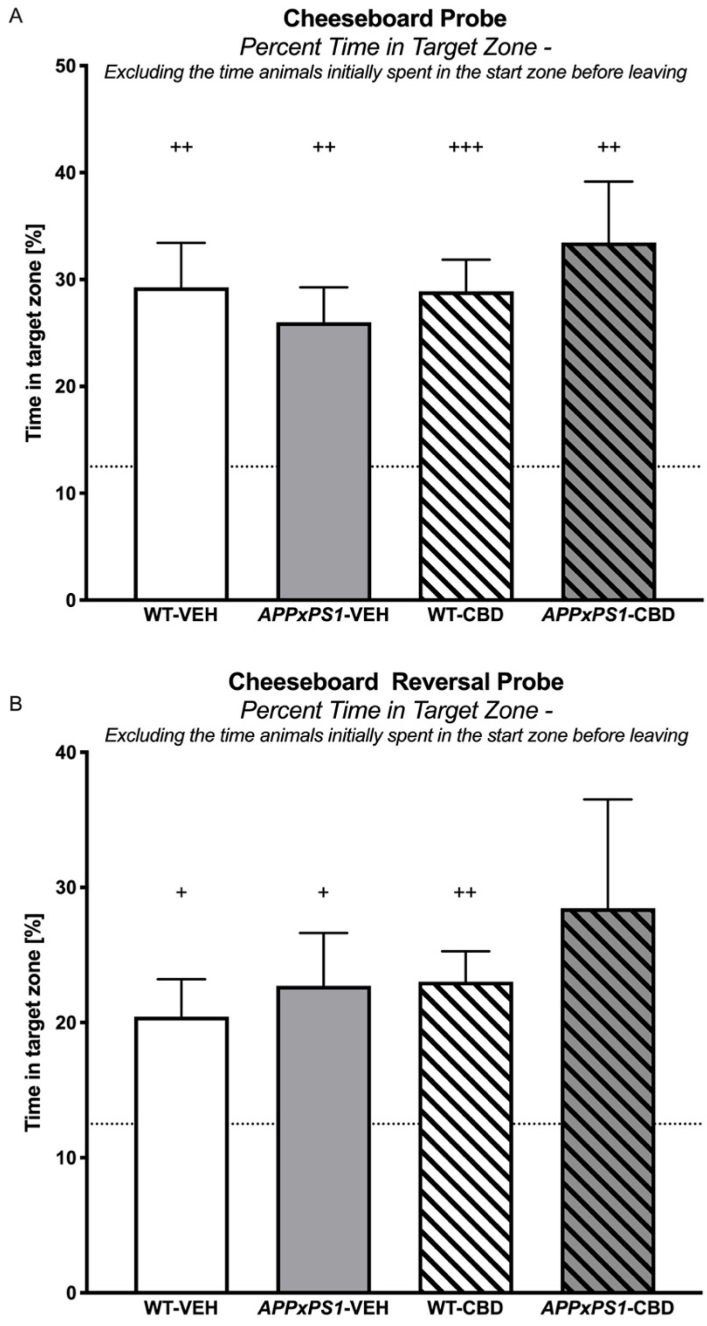

Supplementary Figure S4: Acoustic startle response (ASR) across the first five, middle five and last five blocks of 120dB startle pulses during the prepulse inhibition protocol, i.e. startle habituation. Data for non-transgenic wild type-like (WT) control and double transgenic *APP*$_{Swe}$*/PS1ΔE9* (*APPxPS1*) female mice treated with either vehicle (VEH) or cannabidiol (CBD) are shown as means ± SEM. A significant RM effect of 'time' is indicated by '^' (^^^p < .001).

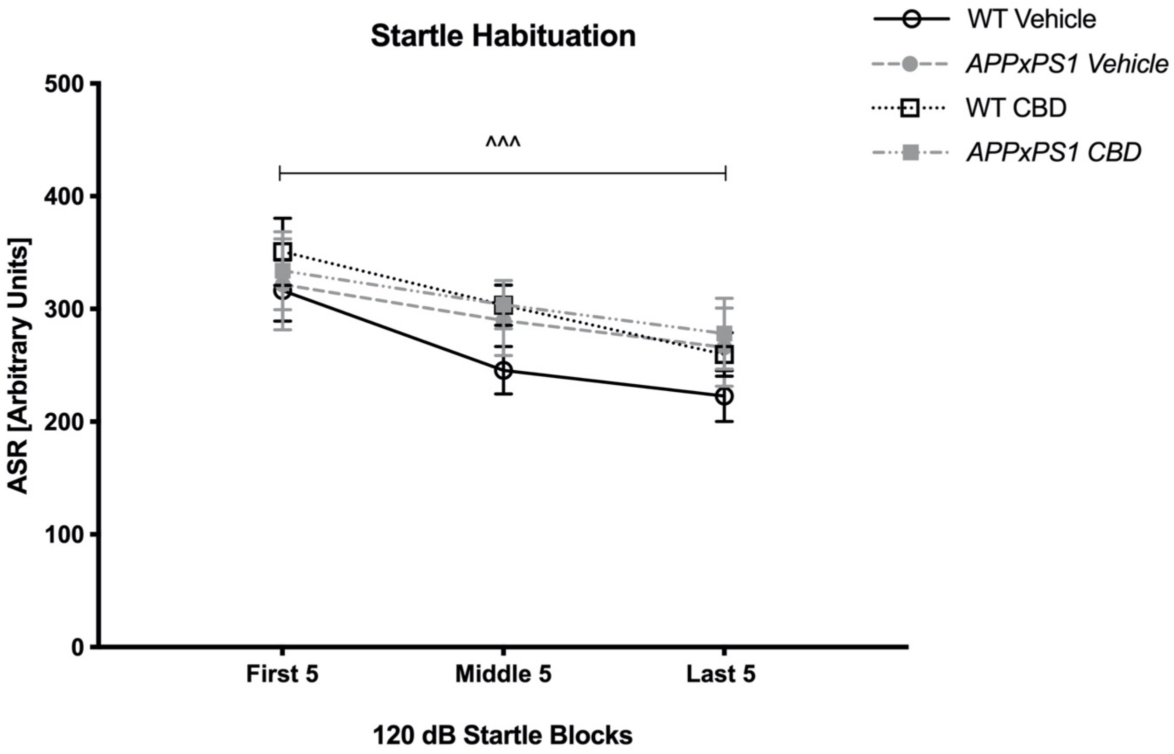

Supplement: Supplementary file 1 [file datasheet1.pdf]
